# Conservation detection dogs: A critical review of efficacy and methodology

**DOI:** 10.1002/ece3.10866

**Published:** 2024-02-15

**Authors:** Beth McKeague, Caroline Finlay, Nicola Rooney

**Affiliations:** ^1^ School of Biological Sciences Queen's University Belfast Belfast UK; ^2^ Conservation Detection Dogs Northern Ireland Comber UK; ^3^ Bristol Veterinary School University of Bristol Bristol UK

**Keywords:** ecology dog, efficacy, methodology, search dog, sniffing dog, standardisation

## Abstract

Conservation detection dogs (CDD) use their exceptional olfactory abilities to assist a wide range of conservation projects through the detection of target specimens or species. CDD are generally quicker, can cover wider areas and find more samples than humans and other analytical tools. However, their efficacy varies between studies; methodological and procedural standardisation in the field is lacking. Considering the cost of deploying a CDD team and the limited financial resources within conservation, it is vital that their performance is quantified and reliable. This review aims to summarise what is currently known about the use of scent detection dogs in conservation and elucidate which factors affect efficacy. We describe the efficacy of CDD across species and situational contexts like training and fieldwork. Reported sensitivities (i.e. the proportion of target samples found out of total available) ranged from 23.8% to 100% and precision rates (i.e. proportion of alerts that are true positives) from 27% to 100%. CDD are consistently shown to be better than other techniques, but performance varies substantially across the literature. There is no consistent difference in efficacy between training, testing and fieldwork, hence we need to understand the factors affecting this. We highlight the key variables that can alter CDD performance. External effects include target odour, training methods, sample management, search methodology, environment and the CDD handler. Internal effects include dog breed, personality, diet, age and health. Unfortunately, much of the research fails to provide adequate information on the dogs, handlers, training, experience and samples. This results in an inability to determine precisely why an individual study has high or low efficacy. It is clear that CDDs can be effective and applied to possibly limitless conservation scenarios, but moving forward researchers must provide more consistent and detailed methodologies so that comparisons can be conducted, results are more easily replicated and progress can be made in standardising CDD work.

## Graphical abstract

Conservation detection dogs (CDD) can be effective and applied to possibly limitless conservation scenarios but moving forward researchers must provide more consistent and detailed methodologies so that comparisons can be conducted, results are more easily replicated and progress can be made in standardising CDD work. This review aims to summarise what is currently known about the use of scent detection dogs in conservation and elucidate which factors affect efficacy.

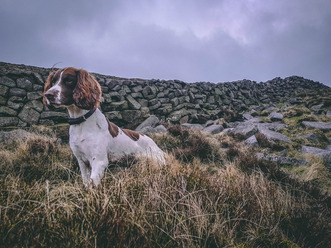

## 1 INTRODUCTION

Domestic dogs (*Canis lupus familiaris*) have worked alongside humans for thousands of years, primarily used for hunting, guarding and even forensic work by the ancient Greeks (Bergström et al., 2020; Helton, 2009; MacKay et al., 2008; Otto et al., 2019; Shields & Austin, 2018; Whitehouse‐Tedd et al., 2021). Even now, dogs support humans by assisting those with disabilities, herding livestock in agriculture, providing protection in law enforcement and military and utilising their sense of smell to find a vast range of substances (Otto et al., 2019; Whitehouse‐Tedd et al., 2021; Woollett et al., 2013). Dogs have searched for numerous targets including accelerants, hazardous chemicals, explosives, illegal drugs, human diseases such as cancer, diabetes and epilepsy, live humans, cadavers and more (Browne et al., 2006; Kokocińska‐Kusiak et al., 2021; Rooney et al., 2019; Whitehouse‐Tedd et al., 2021; Woollett et al., 2013). Within canine scent work, one of the most up and coming areas is that of conservation.

Dogs began working in conservation in the 1890s in New Zealand when they supported efforts to translocate kiwis and kakapos away from areas inhabited by invasive predators (Hill & Hill, 1987). Since then, there has been an almost unlimited scope of their application. Conservation detection dogs (CDD) can perform a variety of tasks like searching for live or dead specimens, nests or burrows and residual scent from hair or urine (Bennett et al., 2022; Helton, 2009; Kokocińska‐Kusiak et al., 2021; Woollett et al., 2013). Additionally, scat surveys have been used for indicating animal presence particularly by using DNA analytical techniques like barcoding (i.e. species identification (Arnot et al., 1993)) and profiling (i.e. identification of an individual organism (Giardina, 2013)) located scats, especially when the scat of different species is visually indistinguishable (Bennett et al., 2022; Helton, 2009; MacKay et al., 2008). CDD use has been documented in 62 countries across over 480 biological species including terrestrial, avian and aquatic mammals, birds, reptiles, amphibians, insects, molluscs, fungi, bacteria and invasive plants (Grimm‐Seyfarth et al., 2021). Seemingly, scent detection dogs have ‘limitless potential’ (Woollett et al., 2013, p. 261) and their application is restricted only by the ‘human imagination’ (Browne et al., 2006, p. 101). They are invaluable, especially during a time when biodiversity is deeply threatened and the risk of extinction faces many species (Ceballos et al., 2017; Díaz et al., 2019).

Given that most animals have olfactory capabilities for navigation and communication (Kokocińska‐Kusiak et al., 2021), why are dogs used most frequently for conservation detection work rather than other species? A key factor is the sheer capacity of canine olfaction. Dogs have up to 250 million olfactory receptors, depending on breed, in comparison to five million in humans (MacKay et al., 2008; Woollett et al., 2013) and can detect odours at concentrations as low as one part per trillion whereas analytical instruments are restricted to parts per billion (Otto et al., 2019). This is due to the unique anatomy of the canine nasal organ and brain (Jenkins et al., 2018; Jezierski et al., 2016; Kokocińska‐Kusiak et al., 2021; MacKay et al., 2008). However, rats, insects and pigs also have the ability to be trained to perform scent discrimination like CDD (Bijland et al., 2013; Cambau & Poljak, 2020; Oh et al., 2015; Teodoro‐Morrison et al., 2014), so why are these species used less frequently?

For conservation work, trainability and capability in the field are required in addition to olfactory acuity (DeMatteo et al., 2019). Canine domestication means that the species has been selected for sociability, motivation and flexibility of learning (Beebe et al., 2016; Helton, 2009; Otto et al., 2019); psychological traits necessary for conducting complex scent work alongside humans. Furthermore, most conservation work takes place outdoors for several hours in varied weather, topographical and vegetation conditions, meaning CDD must be able to traverse great distances, over extended periods of time, while manoeuvring through obstacles. As such, specific physical features are sought when selecting a dog: stamina, agility and resilience to temperature to name a few (Beebe et al., 2016; DeMatteo et al., 2019; Helton, 2009; Otto et al., 2019). These are characteristics seen in many dogs that are rarely found in smaller or less domesticated species.

CDD have been highly beneficial to conservation outcomes. Their use is non‐invasive which protects environmental and wildlife welfare and is preferable to capture‐recapture methods (Browne et al., 2006; Grimm‐Seyfarth & Klenke, 2018; Kerley, 2010; Richards, 2018). Across many circumstances, CDD are faster, can find more samples and cover greater distances during a survey than other methods (Browne et al., 2006; Grimm‐Seyfarth & Klenke, 2018; Kerley, 2010; MacKay et al., 2008; Stanhope & Sloan, 2019). For example, Mathews et al. (2013) found that when comparing humans and CDD during searches for bat carcasses at wind turbine sites, CDD took on average 40 min to conduct a search versus humans taking 2 h and 46 min and CDD found 75% of targets versus humans finding 20%. Furthermore, using CDD can reduce sampling bias as they do not rely on visual information to find targets the way methods like human surveys and camera‐trapping does. Therefore, CDD are more capable of finding obscured samples and those in visually less obvious places (Kerley, 2010; MacKay et al., 2008). Additionally, CDD can play the role of ambassador for conservation work through people's affinity towards dogs (Richards, 2018; Witherington et al., 2017).

However, like any detection tool, disadvantages must also be considered. CDD teams are expensive both in terms of time and money. It takes months, if not years, to train a CDD and its handler along with the monetary cost of training and maintaining the dog through transport, housing, food, etc. (Kerley, 2010; MacKay et al., 2008). Acquiring samples for training can be difficult both practically and legally depending on whether the target species is elusive, endangered, or invasive (Kerley, 2010; MacKay et al., 2008). Moreover, despite generally high efficacy rates, substantial variation occurs (MacKay et al., 2008) which brings CDD reliability into question. Indeed, modern guidelines for conservation methods, such as ‘What Works in Conservation 2021’ by Sutherland et al. (2021) along with governmental protocols for target species searches (Thompson et al., 2020), do not include CDD despite their widespread use, which may be indicative of the concerns around their efficacy. Given that conservation suffers from underfunding (Cozzi et al., 2021; Cristescu et al., 2020), the tool used for a project must be worth the cost.

Hence, this review aims to answer the questions of how, why and to what extent does efficacy varies, as these must be understood to achieve the best results possible when using CDD. To do this, available CDD studies were searched for (*n* = 67) and analysed in light of these questions. A major difficulty facing CDD work is a lack of standardisation across the field (Bennett et al., 2020; Hayes et al., 2018; Johnen et al., 2017; Otto et al., 2019). This is despite efforts made to standardise procedures for the use of scent detection dogs in general (Furton et al., 2010), and for the testing and reporting of CDD work (Bennett et al., 2020; Johnen et al., 2017). At present in CDD literature, terminology for analytical measures is inconsistent (Hayes et al., 2018; Johnen et al., 2017), sample sizes are small leading to low statistical power (Lazarowski et al., 2020; Whitehouse‐Tedd et al., 2021) and according to a systematic review by Johnen et al. (2017), up to 70% of CDD studies report limited training details and almost 25% were considered to be poor quality. All these factors together greatly harm the field's reliability and replicability, which is key to verifying results and improving future research. By assessing efficacy and methodology, issues in the literature can be highlighted, thereby increasing understanding of best practices. In this review, the efficacy of CDD will be investigated across training, testing and operational searches and when searching for different target species. Once efficacy rates have been established, the factors affecting efficacy will be discussed along with how methodological problems may be contributing.

## 2 METHODOLOGY

### 2.1 Selection criteria

Key inclusion criterion informed which studies were selected for the review. The study must have included the use of CDD, whether that be in a laboratory or field environment which also needed to be stated. The performance of the CDD must have been assessed using some type of quantifiable measure. The study also needed to specify what species or at least what group of species the CDDs were trained and tasked with searching for. As this review aimed to assess and critique the efficacy and methodologies of CDD studies across the field, the quality of the chosen papers was not part of the selection criteria in order to fully reflect the current state of the field.

### 2.2 Search

Literature searches took place during April 2022 using the Queen's University Belfast online library (https://www.qub.ac.uk/directorates/InformationServices/TheLibrary/). Search terms used were ‘conservation detection dogs’ and ‘ecology detection dogs’. In total, 67 studies were included in this review based on these searches. It is noted that a more in‐depth search technique involving additional search terms and the use of further databases may have yielded more studies to be included, thus making this a limitation of this study.

### 2.3 Data collection and analysis

Studies with information and results relevant to the selection criteria were collected for analysis and review. Firstly, studies were screened by title, then by abstract, whereby if inspection of the abstract suggested that the study would not meet the necessary criteria for inclusion, it was excluded. Data were extracted from the chosen studies; relating to methodologies (i.e. target scent(s), study setting, the use of blinding, whether comparisons were done with other methods, number of CDDs used and the operational experience of the CDD handler(s) and the results of the CDD performance (i.e. sensitivity and precision; Table 1)).

**TABLE 1:** Glossary of metrics used within the literature review.

Term	Definition	Assumptions	Justification for inclusion or exclusion from use
Sensitivity	‘Proportion of targets found relative to the total number of targets available’ Bennett et al. (2020), p. 5	That the number of targets is known, which may not be the case in field studies	Inclusion: It is a useful tool in training and testing for predicting the probability of detection during operational searches
Precision	‘Proportion of all alerts that are directed towards a true target’ Bennett et al. (2020), p. 5	It is possible to accurately identify the target. In field and operational studies, it is not always possible to visually identify the target (e.g. discriminating a target scat from the scat of another species)	Inclusion: It is an important measure for determining whether the training a CDD has received has provided an accurate scent profile for the target
Specificity	‘Proportion of non‐targets correctly ignored’ Lazarowski et al. (2020), p. 3	That the number of all non‐targets is known. Under field conditions, it is rarely known how many non‐targets are truly present	Exclusion: Due to the issues of using specificity under non‐clinical and uncontrolled conditions, precision can act as a better measure for assessing the dog's ability to discriminate scents in place of specificity
Efficacy	‘The power to produce an effect’ Merriam‐Webster.com Dictionary (2023)	Can be judged based on sensitivity, precision and/or specificity demonstrated during a search	Inclusion: The primary concern of a CDD's performance is whether they are producing the desired effect of their training (i.e. an accurate and reliable search), hence the use and consideration of efficacy

Abbreviation: CDD, conservation detection dogs.

## 3 EFFICACY RATES ACROSS CONTEXTS

When assessing the efficacy of CDD, one must be consistent in which measures are considered to ensure as little bias as possible. However, it can be unclear what a study is measuring and terms like ‘detection rates’ may be used without stating what they quantify regarding the search and dog performance (Hayes et al., 2018; Johnen et al., 2017). Bennett et al. (2020) recommend sensitivity (i.e. proportion of target samples found out of total available), and precision (i.e. proportion of alerts that are true positives), also known as ‘reliability’ or ‘predictive positive value’, as measures to be used for evaluating CDD performance. Sensitivity can investigate performance during training and testing which can then help predict the probability of detection during operational searches, as sensitivity in the field is difficult to ascertain without estimating the total number of targets in an area often using techniques with high margins of error like playback (Bolton et al., 2021). Precision aids in determining the ability of the CDD to distinguish and discriminate the target scent from other odours. Lazarowski et al. (2020) propose measuring sensitivity and ‘specificity’ (i.e. proportion of non‐alerts that are true negatives) in tandem as key to scent detection work. However, they also acknowledge that specificity is often challenging to accurately measure due to the limitless number of distractor scents that may be available during field trials or operational searches, as well as the difficulty of ascertaining that the target scent is completely absent in a natural environment. As such for this review, sensitivity and precision will be the measures of focus (see Table 1). Of the studies reviewed, 46% stated sensitivity rates, though this rate rises to 79% if counting only studies where sensitivity could have been assessed i.e. because the number of potential targets is known (*n* = 39), and 55% provide precision rates.

However, it must be acknowledged that in the field of CDD research and applications, one‐to‐one comparisons of efficacy rates between studies are made difficult by small sample sizes (Lazarowski et al., 2020; Whitehouse‐Tedd et al., 2021) and lack of detail about the studies (Johnen et al., 2017). Indeed, of the 67 studies identified for this review, the sample sizes of CDDs ranged from 1 to 20, and 6% (*n* = 4) did not report this clearly (see Table 2). For the purposes of detecting statistically significant differences and effect sizes, these sample sizes are far too low. But in many ways, this is the nature of the field given the costs and practicalities associated with training and maintaining a CDD (Kerley, 2010; MacKay et al., 2008) and it is unlikely that future studies with vastly larger sample sizes will be produced any time soon. Therefore, there is a need to assess the current state of research in the field, comparing specific comparable measures where available (Table 1) while also acknowledging limitations.

**TABLE 2:** Summary of literature reviewed that investigates CDD efficacy rates.

Citation	Target scent (material)	Sensitivity (trial setting)	Precision (trial setting)	Study type	Blinding	Compared to	No. of dogs used (trained)	Operational experience of CDD in time
Arandjelovic et al. (2015)	Cross River gorilla (*Gorilla gorilla diehli*) (scat)	N/A	NS *0.97 samples found per team day	F	N/A	Human (0.43 samples found per team day)	3	NS
Arnesen and Rosell (2021)	Asian longhorn beetle (*Anoplophora glabripennis*), Citrus longhorn beetle (*Anoplophora chinensis*)	80.4% (lab), 87.8% (semi‐field), 95.3% (standardised field)	NS	TT	Double	N/A	2	2–3 years
Arnesen et al. (2020)	Rock ptarmigan (*Lagopus muta*) (scat)	66.7% (lab)	NS	TT	Double	N/A	4 (5)	NS
Arnett (2006)	Bat species	71% & 81% (field efficiency trials across two locations respectively)	NS	Both	NS	Human (42% & 14% across two locations respectively)	2	NS
Bearman‐Brown et al. (2020)	West European hedgehog (*Erinaceus europaeus*)	N/A	NS *20 samples detected	F	N/A	Infra‐red thermal camera (19 samples detected), Spotlight survey (8 samples detected)	1	NS
Becker et al. (2017)	Cheetah (*Acinonyx jubatus*) (scat)	N/A	NS *50 samples detected	F	N/A	Spoor surveys (0 samples detected)	2	9 years (collective)
Beckmann (2006)	Black bear (*Ursus americanus*), Grizzly bear (*Ursus arctos horribilis*), Cougar (*Puma concolor*), Grey wolf (*Canis lupus*) (scat)	N/A	98.6%	F	N/A	N/A	4	NS
Bolton et al. (2021)	Storm petrel (*Hydrobates pelagicus*), Manx shearwater (*Puffinus puffinus*)	86.5% (field 1), 62.6% (field 2) *Assessed using playback	67%–100% (lab 1), 50%–100% (lab 2), 50%–95% (lab 3), 97.5% (field 1)	Both	Double	N/A	2	NS
Brook et al. (2012)	Javan rhinoceros (*Rhinoceros sondaicus*) (scat)	N/A	NS *22 samples detected	F	N/A	N/A	2	NS
Browne et al. (2015)	Tuatara (*Sphenodon punctatus*), Marlborough green gecko (*Naultinus manukanus*), Forest gecko (*Mokopirirakau granulatus*)	NS	61.6%–82.7% (lab, gecko), 76.2%–93.7% (lab, tuatara)	TT	Double (tester present)	N/A	13 (20)	3 days–8 years (scent training)
Cablk and Heaton (2006)	Desert tortoise (*Gopherus agassizii*)	91% (surface overall), 86% (burrow 1 overall), 98% (burrow 2 overall)	27% & 73% (surface, dog 1 and dog 2 respectively), 68% (burrow 1 overall), 56% (burrow 2 overall)	TT	Double	N/A	2 (5)	NS
Chambers et al. (2015)	Bat species (roosts, guano)	78.6% (controlled field, guano), 28.6% (controlled field, roost)	NS	TT	NS	N/A	2	NS
Clare et al. (2015)	Bobcat (*Lynx rufus*) (scat)	N/A	64% *59 samples out of 92 genetically amplified	F	N/A	Camera‐trapping (129 detection events)	2	NS
Cozzi et al. (2021)	Wolf (scat)	N/A	84.38% *43 scats indicated on, 32 genetically amplified, 27 found to be wolf **83% of valleys surveyed occupied	F	N/A	Camera‐trapping (66% of valleys surveyed occupied), Audio recorders (17% of valleys surveyed occupied)	3	NS
Cristescu et al. (2015)	Koala (*Phascolarctos cinereus*) (scat)	97% (field, leash), 100% (field, off leash)	NS *23 locations with scat identified in field trial	Both	Double	Human (15 locations with scat identified in field trial)	1	NS
Davidson et al. (2014)	Cougar (scat)	N/A	68.7% *272 scats indicated on, 249 genetically amplified, 171 targets found	F	N/A	N/A	2	NS
Dematteo et al. (2009)	Bush dog (*Speothos venaticus*)	N/A	NS *22 locations identified	F	N/A	N/A	1	NS
DeMatteo et al. (2014)	Jaguar (*Panthera onca*), Puma, Ocelot (*Leopardus pardalis*), Oncilla (*Leopardus tigrinus*) (scat)	N/A	76% *447 scats confirmed as one of target species, 22 identified as a combination, 45 failed to amplify, 40 only prey species confirmed	F	N/A	N/A	1	NS
Domínguez del Valle et al. (2020)	Bat and bird species	77.3% (field)	NS	TT	Single	Human (21.5%)	3	NS
Duggan et al. (2011)	Franklin's ground squirrel (*Poliocitellus franklinii*)	NS	44%–67% (F, indication only, 1 dog to both), 59%–83% (F, indication and behaviour change, 1 dog to both) *Equivalent to two daily livetrapping surveys	Both	Double	Livetrapping *10 samples trapped across 40 sites	2	NS
Engeman et al. (2002)	Brown tree snake (*Boiga irregularis*)	61% (field 1), 64% (field 2)	NS	TT	Double	N/A	NS	NS
Fukuhara et al. (2010)	Indian mongoose (*Herpestes auropunctatus*) (scat)	92% (TT: field)	98% (TT: field), 92% (F)	Both	Double	N/A	2	>1 year
Fukuhara et al. (2022)	Indian peafowl (*Pavo cristatus*) (eggs)	100% (TT: field)	100% (TT: field) *423 nests located (F)	Both	Double	N/A	3	NS
Furtado et al. (2008)	Jaguar (scat)	N/A	50%–81%	F	N/A	N/A	NS	NS
Glen et al. (2016)	Feral cat (*Felis catus*)	N/A	NS *Detections in 5 out of 9 cells	F	N/A	Camera‐trapping *Detections in 4 out of 9 cells	2	5–7 years
Goodwin et al. (2010)	Spotted knapweed (*Centaurea stoebe*)	NS *Overall accuracy 86%	94% (field)	TT	Double	Human (100% precision, 59% accuracy)	3	1–6 years
Grimm‐Seyfarth et al. (2019)	Eurasian otter (*Lutra lutra*), American mink *(Neogale vison*) (scat)	100% (lab)	85% (lab, mink), 97% (lab, otter) *8.8–12.6 scats/h found (outside transect‐transect, F)	Both	Double	Human (45%–100% sensitivity & 63%–89% precision (beginners to experts, lab), 5.5–8.5 scats/h found (outside transect‐transect, field))	4	NS
Hansen and Winje (2021)	Sheep (*Ovis aries*) (remains)	23.8% (TT: field)	NS *132 carcass remains found (F)	Both	NS	Human (2.5% (TT: field), 76 carcass remains found (F))	16	NS
Harrison (2006)	Bobcat (scat)	N/A	89% (DNA)–91% (CSD) *78 scats detected, 63 DNA identified, 68 tested through CSD	F	N/A	Hair snare (1 target sample), Scent station (0 target samples), Camera‐trapping (5 target detections)	1	NS
Hatlauf et al. (2021)	Golden jackal (*Canis aureus*) (scat)	N/A	73% *34 scats detected, 26 genetically amplified, 19 target samples found	F	N/A	Bioacoustic stimulation (45% response rate)	2	NS
Hofmann et al. (2021)	Cheetah (scat)	45%, 75%, 93.3% (field:dog only, human vs. dog, ‘effective transect area’)	100% (field, human vs. dog)	TT	Double (tester present)	Human (22% sensitivity, 56% precision)	1	6 years
Hollerbach et al. (2018)	Eurasian lynx (*Lynx lynx*) (scat)	N/A	30.8% *169 samples detected, 130 genetically amplified, 52 target samples **Target presence detected in 47.8% of grid cells (21/44)	F	N/A	Camera‐trapping (Target presence detected in 36.3& of grid cells (16/44))	2	NS
Hoyer‐Tomiczek et al. (2016)	Asian longhorn beetle	75%–88.1% (field), 85%–92.6% (lab)	NS	TT	Double	N/A	18	Several months‐6 years
Jean‐Marie et al. (2019)	Hermann tortoise (*Testudo hermanni*)	NS *86 targets found	NS	TT	NS	Human (30 targets found)	6	NS
Kapfer et al. (2012)	Eastern box turtle (*Terrapene carolina carolina*)	N/A	NS *25 targets found across 2 days	F	N/A	Human (22 targets found across 316.5 h)	NS	NS
Kretser et al. (2016)	Moose (*Alces alces*) (scat)	N/A	69% *195 scats detected total (191 by CDD, 4 by orienteers), 134 target samples	F	N/A	N/A	2	NS
Liczner et al. (2021)	Bumble bee species (nests)	NS	NS	TT	NS	N/A	3	NS
Long et al. (2007)	Black bear, Fisher, Bobcat (scat)	N/A	NS *728 and 868 scats detected in 2003 and 2004 respectively **Black bears detected at 57.1% of sites, fishers detected at 61.3% of sites, bobcats detected at 12.5% of sites	F	N/A	Camera‐trapping (black bears detected at 23.7% of sites, fishers detected at 19.6% of sites, bobcats detected at 5.4% of sites), Hair snare (black bears detected at 9.5% of sites, no fishers or bobcats detected)	5	NS
Mathews et al. (2013)	Bat species	75% (field)	NS	TT	Double	Human (20%, field)	2	NS
Matthew et al. (2021)	Giant bullfrog (*Pyxicephalus adspersus*)	87% (lab)	84% (lab)	TT	Double	N/A	1	NS
McGregor et al. (2016)	Feral cat	N/A	NS *35 targets captured over 476 person‐hours	F	N/A	Leg‐hold trapping (19 targets captured across 1112 trap nights)	2	NS
Mosconi et al. (2017)	Hermit beetle (*Osmoderma eremita*)	55%–84% (field)	NS	TT	Double	N/A	1	NS
Needs et al. (2021)	Tall Daisy (*Brachyscome diversifolia*)	100% (lab)	85%–100% (lab)	TT	Double (tester present)	N/A	8	NS
O'Connor et al. (2012)	Bumble bee species (nests)	79% (TT: controlled field)	75% (TT: controlled field) *10 nests found (F)	Both	Double	Human (10 nests found (F))	1	NS
Oldenburg et al. (2016)	Eurasian otter (*lutra lutra*) (scat)	NS	100% (lab 1 & 2) 70% (lab 3)	TT	Double	N/A	1	NS
de Oliveira et al. (2012)	Red brocket (*Mazama americana*), Grey brocket (*Mazama gouazoubira*), Small red brocket (*Mazama Bororo*) (scat)	29% (field)	NS	TT	NS	Human (0%)	1	NS
Orkin et al. (2016)	Western black crested gibbon (*Nomascus concolor*), Indochinese grey langurs (*Trachypithecus crepusculus*), Stump‐tailed macaques (*Macaca arctoides*) (scat)	N/A	81% (including unidentified), 92% (excluding unidentified)	F	N/A	Human (45% (including unidentified), 76% (excluding unidentified))	1	NS
Paula et al. (2011)	Bat and bird species	96% (field)	100% (field)	TT	Double	Human (9% sensitivity, field)	1	NS
Petroelje et al. (2021)	Prey remains of Grey wolves, Black bear, Coyote (*Canis latrans*), Bobcat	N/A	NS *149 clusters with targets found	F	N/A	Human (19 clusters with targets found)	3	NS
Reed et al. (2011)	Mountain lion, Bobcat, Domestic cat, Red fox (*Vulpes vulpes*), Grey fox (*Urocyon cinereoargenteus*), Kit fox (*Vulpes macrotis*) (scat)	68% (field, dog 1), 77% (field, dog 2)	NS	TT	Single	N/A	2	NS
Reindl‐Thompson et al. (2006)	Black‐footed ferret (*Mustela nigripes*)	71%–100%% (field 1), 57%–100% (field 2)	100% (field 1 & 2)	TT	Double	Spotlight survey (100% precision)	2	NS
Reynolds et al. (2021)	Koloa (*Anas wyvilliana*) (carcasses infected with avian botulism)	82% (TT: field 1), 77% (TT: field 2 timed)	NS	Both	Double	Human (70% (TT: field 1), 39% (TT: field 2 timed), All‐terrain vehicle (ATV) (40%, TT: field 1))	4	<1–4 years
Rolland et al. (2007)	North Atlantic right whale (*Eubalaena glacialis*) (scat)	N/A	NS *97 samples detected	F	N/A	Human (30 samples detected)	3	NS
Rutter, Mynott, et al. (2021)	Alpine stonefly (*Thaumatoperla alpina*)	100% (TT: lab)	87.5% (TT: lab)	Both	Double	N/A	4	NS
Sentilles et al. (2021)	Pyrenean brown bear (*Ursus arctos*) (scat)	N/A	100% *68% of 750 scats collected total	F	N/A	Human (32% of 750 scats collected total)	1	NS
Smallwood et al. (2020)	Bat and bird species	96% (field, bats), 90% (field, birds)	NS	TT	Double	Human (6% bats, 30% birds)	2	NS
Smith et al. (2003)	San Joaquin kit fox (*Vulpes macrotis mutica*) (scat)	NS	100% (F)	Both	Double	NS	4 (7)	<1–2 years
Statham et al. (2020)	Blunt‐nosed leopard lizard (*Gambelia silus*) (scat)	N/A	82.4%	F	N/A	N/A	3	NS
Stevenson et al. (2010)	Eastern indigo snake (*Drymarchon couperi*)	91% (TT: field)	NS	Both	Double	N/A	1	NS
Thomas et al. (2020)	Black‐tailed antechinus (*Antechinus arktos*)	NS	100% *31 targets indicated across three deployments	F	N/A	Camera‐trapping (74 targets detected over 240 trap nights), live capture (11 target captures over 2400 trap nights)	1	NS
Thompson et al. (2020)	Bilby (*Macrotis lagotis*) (scat)	98.9% (field)	100% (field)	TT	Double	Human (6.7% sensitivity, 100% precision)	1	NS
Thompson et al. (2012)	Fisher (*Pekania pennanti*) (scat)	N/A	55.4% *241 scats detected, 184 genetically amplified, 104 target samples	F	N/A	N/A	NS	NS
Vesely (2008)	Kindcaid's Lupine (*Lupinus sulphureus* subsp. *kincaidii*)	98.8% (field)	97.5% (field)	TT	Single	N/A	3	2–8.5 years
Vynne et al. (2011)	Maned wolf (*Chrysocyon brachyurus*), Puma, Jaguar, Giant anteater (*Myrmecophaga tridactyla*), Giant armadillo (*Priodontes maximus*) (scat)	N/A	71.1% *2683 scats detected, 2250 collected, 1600 target samples	F	N/A	N/A	3	NS
Wasser et al. (2004)	Grizzly bear, Black bear (scat)	N/A	NS	F	N/A	N/A	5	NS
Waters et al. (2011)	Bumble bee species (nests)	100% (TT: controlled field)	100% (TT: controlled field)	Both	Double	N/A	1	NS
Witherington et al. (2017)	Loggerhead sea turtle (*Caretta caretta*), Green sea turtle (*Chelonia mydas*) (eggs)	NS	100% (field 1), 98.8% (field 2)	TT	NS	N/A	1	NS

*Note*: * indicates additional detail/context regarding methodology and how results were reported in the respective paper study. ** indicates further information regarding methods and results reporting if/when several different techniques were used by the respective paper study.

Abbreviations: CDD, conservation detection dogs; F, field work; NS, not stated; N/A, not applicable; TT, training/testing.

In controlled training and testing trials, the ability to find present targets accurately appears to vary greatly. For insects like beetles, bumble bees and stoneflies, reported sensitivity ranged from 55% to 100% and precision from 75% to 100% with the use of targets like nest material, infested wood, or larvae (Arnesen & Rosell, 2021; Hoyer‐Tomiczek et al., 2016; Mosconi et al., 2017; O'Connor et al., 2012; Rutter, Mynott, et al., 2021; Waters et al., 2011). For plant species, rates were high with 99% to 100% sensitivity and 85% to 100% precision (Goodwin et al., 2010; Needs et al., 2021; Vesely, 2008). Work with reptiles and amphibians reported rates of between 61% to 98% for sensitivity and 27% to 100% for precision (Browne et al., 2015; Cablk & Heaton, 2006; Engeman et al., 2002; Matthew et al., 2021; Stevenson et al., 2010; Witherington et al., 2017). CDDs detecting carcasses of birds and bats on windfarms were reported to show sensitivities between 71% and 96% (Arnett, 2006; Domínguez del Valle et al., 2020; Mathews et al., 2013; Smallwood et al., 2020). Searching for bird species through scat, carcasses, or eggs has resulted in sensitivity rates between 62.6% and 100% with precision reported between 50% to 100% (Arnesen et al., 2020; Bolton et al., 2021; Fukuhara et al., 2022; Reynolds et al., 2021). However, the study by Arnesen et al. (2020), where dogs searched for rock ptarmigan (*Lagopus muta*) scat in lab conditions, had three dogs out of four perform no better than chance and none of the dogs or handlers had any previous experience of training for CDD work.

Mammals are by far the most common animals searched for in published studies. For small mammals, sensitivity during training and testing contexts ranged from as low as 29% to as high as 100% with 44% to 100% precision (Chambers et al., 2015; Cristescu et al., 2015, 2019; Duggan et al., 2011; Fukuhara et al., 2010; Grimm‐Seyfarth et al., 2019; Reindl‐Thompson et al., 2006; Thompson et al., 2020). Regarding the 29% sensitivity in Chambers et al. (2015) for finding natural bat roosts, this was during a search for both natural bat roosts and suspended bags of guano where guano was the original trained target. This could have caused the CDD to have a preference for the guano samples (i.e. on which they had been imprinted and trained) over the bat roosts which were novel. Indeed, sensitivity was 79% on guano bags alone, and increased to 77% for finding bat roosts, when only searching for bat roosts not in the presence of guano bags. The concepts of using different samples in training versus testing, generalisation of CDD to non‐trained targets and the effects of odour concentration in search performance are elaborated on further in the Training section.

For larger mammals, sensitivity rates during training and testing of between 23.8% for sheep remains and 93.3% for cheetah scat are reported (de Oliveira et al., 2012; Hansen & Winje, 2021; Hofmann et al., 2021; Reed et al., 2011) with Hofmann et al. (2021) demonstrating 100% precision on cheetah scat. Although 23.8% sensitivity for CDD seems low, this was compared to the 2.5% sensitivity of human searchers looking for the same carcasses (Hansen & Winje, 2021). Improvements in detection by even small proportions can be hugely beneficial as conservation projects often rely on methods with overall low detection rates (Mathews et al., 2013). These examples demonstrate little pattern regarding the target species when it comes to success during training and testing except for greater variation with mammal targets which could be due to a few things: an inherent issue with the target odours, variation in the quality of the studies, or the simply greater number of papers in that area (i.e. of 67 studies reviewed: 44 on mammals, eight on reptiles, seven on birds with three of these overlapping with mammal studies, seven on invertebrates, three on plants, one on amphibian) (see Table 2).

CDD efficacy should be evaluated during training and testing rather than waiting until operational searches to assess performance, however, many published studies simply investigate whether CDD can discriminate the target odour in a simple controlled trial and do not progress to testing the CDD in a field environment under operational conditions. Indeed, of the 67 studies examined in this review, 37% focus only on training and testing (*n* = 25), 42% assess solely field performance (*n* = 28) and 21% look at both (*n* = 14). Of those studies that measure training and testing performance, 33% conduct their experiments in purely lab‐based or controlled field conditions. Moreover, seemingly obvious statistics are sometimes stated such as strong positive correlations between CDD alerts and true positives (Bolton et al., 2021; Oldenburg et al., 2016) which simply means that the dog is doing what it has been trained to do; an unsurprising result given the decades of effective scent detection work performed by canines. This breakdown shows a skew towards laboratory‐based and controlled trials that do not translate into assessing fieldwork capabilities or improving methodological practices.

Sensitivity and precision rates within fieldwork vary similarly to those of training and testing. Although most operational windfarm mortality searches did not report precision, Paula et al. (2011) achieved rates of 100% meaning all indications were true positives. Of studies assessing performance in the field, scat surveys of mammals are the most prevalent with precision rates of between 30.8% to 100% (Beckmann, 2006; Clare et al., 2015; Cozzi et al., 2021; Davidson et al., 2014; DeMatteo et al., 2014; Furtado et al., 2008; Harrison, 2006; Hatlauf et al., 2021; Hollerbach et al., 2018; Kretser et al., 2016; Orkin et al., 2016; Sentilles et al., 2021; Smith et al., 2003; Statham et al., 2020; Thompson et al., 2012; Vynne et al., 2011). Low rates of precision may occur as it can be difficult for the handler to accurately identify scats visually which can lead to them accidentally rewarding indications on non‐target scats (i.e. false positives) hence reinforcing and leading to a subsequent increase in their frequency. Additionally, CDD may be correctly alerting and DNA barcoding and profiling of the scat can be wrong due to contamination from non‐target species resulting from coprophagy, urination, and contact with saliva (DeMatteo et al., 2018). Furthermore, both Hollerbach et al. (2018) and Kretser et al. (2016) used CDDs which had also been trained to indicate on other targets as part of previous work. Training CDD to detect multiple species with overlapping habitats can lead to indications on all targets. As such, most of the false positives in these studies were for the previously trained targets which although classified as a false positive in the context of the study, is not a false positive in the context of the dog's training.

Unfortunately, even while assessing the ability of CDD using these set measures, not every study reports results clearly enough to make inferences. Sometimes, the number of targets found is the only measure reported due to budget constraints, being unable to verify true positives in the field (e.g. small mammals hiding or denning in inaccessible places (Thomas et al., 2020)), or simply a lack of information given within the study itself (Arandjelovic et al., 2015; Bearman‐Brown et al., 2020; Becker et al., 2017; Brook et al., 2012; Dematteo et al., 2009; Glen et al., 2016; Jean‐Marie et al., 2019; Kapfer et al., 2012; Liczner et al., 2021; Long et al., 2007; McGregor et al., 2016; Petroelje et al., 2021; Rolland et al., 2007; Thomas et al., 2020; Wasser et al., 2004, 2012). Although these results are still valuable for comparisons with other methods and establishing species presence, without any information on error rates, it cannot be determined whether the CDD is performing efficiently or if the authors are merely reporting successes and ignoring mistakes (i.e. are the dogs actually indicating on a number of species including species unwanted by the study (false positives) or are the finds even being verified in the field and the dog's indication taken as writ?).

Despite this, it is clear that across training, testing and operational tasks, CDDs perform generally well and much better than other methods with CDDs outperforming humans and other analytical tools in 91% of the 34 studies analysed where comparisons were made (see Table 2, Columns 3, 4 and 7), excluding select cases: bumble bee nest detection where performance was equivalent to humans (O'Connor et al., 2012) and rhinoceros scat searches where the size of the scat means CDDs do not provide a distinct advantage over the standard method (Brook et al., 2012). However, this review has established that sensitivity and precision rates still vary by a large margin across the studies regardless of target species and search context. So, the question remains, what drives the variation in CDD efficacy?

## 4 FACTORS AFFECTING EFFICACY AND METHODOLOGICAL ISSUES

### 4.1 Training

Training is the foundation of CDD performance with several stages including imprinting, indication, search tasks and discrimination trials (DeMatteo et al., 2019). Each has the potential to affect efficacy. In the context of scent detection dogs, imprinting is the process of familiarising the CDD with the target odour (Mosconi et al., 2017). Given the sensitivity of the canine nose, sample handling during training must be conducted with care (Kokocińska‐Kusiak et al., 2021; Lazarowski et al., 2020). Subtle aspects of sample preparation can lead to the dog learning that another odour is paired with the reward rather than the target itself (Guest et al., 2020). Papers often provide only limited information on sample storage and handling so no inference can be made on whether this affected efficacy. Indeed, issues identified regarding sample use include sample contamination with human scent (Arnett, 2006) or other non‐target scents (Vynne et al., 2011), poor decontamination procedures like running under hot water rather than sterilisation of sample storage devices, dog saliva touching sample containers (Rutter, Howell, et al., 2021a, 2021b), and urination and/or defecation by dogs during searches (Browne et al., 2015; DeMatteo et al., 2018; Heaton et al., 2008) which poses a threat to samples and ecosystems (Whitehouse‐Tedd et al., 2021). Goss (2019) provides detailed information on the importance of proper sample storage and which materials are and are not appropriate for use as storage devices. Furthermore, a review of detection dog work suggests that over 20% of studies may have used the same samples across training and testing (Johnen et al., 2017) which means the dog may have learnt the specific samples rather than the target odour profile (Stanhope & Sloan, 2019).

Given that CDD are biological systems, their olfactory function is subject to many influences (Kokocińska‐Kusiak et al., 2021). Factors linked to reductions in olfaction capability include older age, use of certain pharmaceuticals, diseases, dehydration, diet and nutrition, activity levels and environmental influences like temperature, humidity and precipitation (Gutzwiller, 1990; Hayes et al., 2018; Jenkins et al., 2018; Kokocińska‐Kusiak et al., 2021). There is simply no way to know if any internal variables may have played a role in CDD efficacy if details are missing about the dogs used and their care. Furthermore, the target odour that a CDD has been trained to find can also affect operational search efficacy, as it is unclear whether CDD search for complete odour signatures or simply components of the target odour that are present across samples and conditions (Johnen et al., 2017). Indeed, CDD are very capable of generalising from low scent profiles during training to full specimens in the field and vice versa (Dematteo et al., 2009; Oldenburg et al., 2016; Rutter, Mynott, et al., 2021). However, depending on the samples used to train the dog, different errors may be made in the field. For example, if trained on low concentrations of odour then CDD may alert where no visual sample can be found due to residual scent from past specimen presence, which is an issue that Duggan et al. (2011) faced when searching for Franklin's ground squirrel. Alternatively, smaller samples may be missed more frequently than larger samples as seen with Goodwin et al. (2010) in searches for spotted knapweed. This can occur depending on whether training involved only high odour concentration samples or failed to simulate any aspect of search environments through field tests and discrimination training, meaning the sample can be masked by non‐target scents from wildlife or the environment (Gutzwiller, 1990).

Indication or alerting is how a CDD informs a handler that they have found a target through a distinct and consistent change in behaviour (Johnen et al., 2017). Indications can be passive (i.e. no interaction with target) or active (i.e. body contact with target) depending on the needs of a project. Passive indication is recommended for CDD work to protect sample integrity and the safety of both the dog and wildlife (DeMatteo et al., 2019; MacKay et al., 2008; Matthew et al., 2021; Mosconi et al., 2017). However, details and definitions of CDD indications are regularly omitted in the literature. Furthermore, some authors report changes of behaviour (COB; i.e. notable shifts in CDD behaviour that suggest the dog has found or is tracking a scent) or partial indications as a suitable criteria for a true positive (Cablk & Heaton, 2006; Clare et al., 2015; Duggan et al., 2011; Hoyer‐Tomiczek et al., 2016; Stevenson et al., 2010) which is far more subjective and open to interpretation and unable to be standardised, thus affecting efficacy rates (Lazarowski et al., 2020).

Several types of search tasks can be used when training and testing CDD efficacy (Helton, 2009). Multiple‐choice tasks are where the CDD has the option to investigate multiple containers and is rewarded if they alert on the correct one (Gadbois & Reeve, 2016; Helton, 2009). These can simulate exposure to different scents available in the field and also facilitate discrimination training which is key to ensuring CDD are exposed to commonly encountered scents that should be ignored in favour of the target odour (Arnesen & Rosell, 2021; Bennett, 2015; Boroski & Oliver, 2018; Gadbois & Reeve, 2016; Mosconi et al., 2017; Statham et al., 2020). However, they also provide more sensory interference for the dog and can cause preferences for specific container positions which makes assessing true odour discrimination and indication performance more difficult (Gadbois & Reeve, 2016; Lazarowski et al., 2020). Alternatively, yes/no or go/no‐go tasks involve presenting the dog with a singular sample and rewarding if they make the correct choice in alerting or ignoring (Gadbois & Reeve, 2016; Helton, 2009). These allow for a clear examination of where the dog may be making mistakes and whether they are making choices more liberally (i.e. more false positives) or conservatively (i.e. more false negatives; Gadbois & Reeve, 2016). However, requiring the dog to have greater response inhibition during these tasks can make them needlessly challenging (Lazarowski et al., 2020). Yes/no tasks have been recommended for CDD as they make the calculation of specificity and measures of accuracy comparable (Gadbois & Reeve, 2016; Johnen et al., 2017), but multiple‐choice tasks are commonly seen in the literature. Although this method has benefits, it often lacks details on dog performance which can help estimate and explain field efficacy rates.

A vital factor for ensuring efficacy results are reliable is blinding (Elliker et al., 2014). Single blinding is done to ensure the dog is using olfaction rather than memory to find the target, but double blinding is preferred where both the handler and tester also do not know where the target is (Boroski & Oliver, 2018; Johnen et al., 2017; Lazarowski et al., 2020; Stanhope & Sloan, 2019). This avoids the ‘Clever Hans effect’ which is an example of a horse seemingly being able to count but instead was reading human behaviour to determine when the correct response was given in order to receive a reward (Lazarowski et al., 2020; Sebeok & Rosenthal, 1981). Domesticated animals like dogs are highly skilled at reading human behaviour (Lazarowski et al., 2019), so even in cases where the handler or tester knows the target location and believes that efficacy will be unbiased due to the dog ignoring them for the most part (Browne et al., 2015; Domínguez del Valle et al., 2020; Needs et al., 2021; Vesely, 2008), they may still unconsciously and unintentionally signal the location of the target to the CDD. Indeed, Kardish et al. (2015) found that within ecological, evolutionary and behavioural research, only 13.3% of studies susceptible to observer bias, reported the use of blinding. In our own review we found that 82% of the studies described in Table 2 where blinding could apply (i.e. where training/testing took place; *n* = 39) used blinding, with 91% of these being double blinding and 9% single‐blinding. In other cases, it is either unreported or more worryingly not being conducted at all, though it is a comfort to see the rates of blinding higher than previous studies suggested.

### 4.2 CDD selection and the handler

Although CDD are used as a tool for detection, unlike analytical devices each individual dog will differ which means the selection criteria of CDD for efficacy is vital. There is little doubt that all dogs with a functioning sense of smell can detect a target that emits odour (Woollett et al., 2013). This has been demonstrated with pet dogs and their owners that have been trained to perform scent discrimination and search tasks for novel odours similar to CDD teams (Rutter, Howell, et al., 2021a, 2021b). However, the breed of CDD is often considered influential in achieving the biological and psychosocial traits necessary for fieldwork. Breeds that have been historically selected for their scent abilities are frequently used under the belief that they will inherently perform well (Lazarowski et al., 2020). However individual differences can affect efficacy (Jamieson et al., 2017). Across CDD literature 128 breeds of dogs have been used and minimal differences found in suitability (Grimm‐Seyfarth et al., 2021). Furthermore, the assumption that brachycephalic breeds will perform worse is unverified with pugs outperforming German Shepherds in scent discrimination tests (Hall et al., 2015), although their ability to physically endure under field conditions is untested.

More important than breed‐specific differences is individual personality. No standardised measures for conducting personality testing exist and it is unknown when in the dog's life cycle their ability to work can be determined (Beebe et al., 2016). Indeed, wastage (i.e. failing training) is a major problem in breeding for CDD as the dog may be unsuited to conservation work (Byosiere et al., 2019). The essential characteristics for CDD are high play and/or food drive, high hunt drive and low prey drive (Bearman‐Brown et al., 2020; Beebe et al., 2016; DeMatteo et al., 2019; Helton, 2009; Jamieson et al., 2017; Smith et al., 2003; Statham et al., 2020; Vynne et al., 2011; Wasser et al., 2004; Willcox et al., 2019). However, most assessments of these traits rely on the subjective view of whoever chooses the dog (Beebe et al., 2016). Moreover, dogs are biological systems and there will always be an amount of variability in performance based on countless internal and external factors throughout their development (Kokocińska‐Kusiak et al., 2021; Woollett et al., 2013).

CDDs must work as a team alongside a human handler who oversees searches, verifies finds and reinforces training. As such, the handler also plays a crucial role in CDD outcomes. Similar to dogs, specific skills and traits must be demonstrated to become a handler: ability to direct a search by assessing where the dog has yet to investigate, understanding of animal behaviour, learning and scent theory, attention to detail, consistency and endurance for working in field conditions (Beebe et al., 2016; Boroski & Oliver, 2018; DeMatteo et al., 2019; Helton, 2009). Handlers can both positively and negatively influence dog performance. The handler's beliefs about how a search will go or the dog itself (Lit et al., 2011), the handler's behaviour during a search regarding possible finds, the handler's level of experience (Jamieson et al., 2018b; Lazarowski et al., 2019, 2020) and their personality can all affect the dog's behaviour (Hayes et al., 2018; Jamieson et al., 2018a; MacKay et al., 2008). Furthermore, the bond between a CDD and handler matters for search performance (Bennett, 2015; Mosconi et al., 2017; Otto et al., 2019). Dogs working with an unfamiliar handler, display more stress‐related behaviours and have reduced search efficacy, if they will even search at all (Jamieson et al., 2018b; Springer, 2011).

### 4.3 Search environment and method

Various elements of a search including the area and methods used, also play a role in efficacy. The environment is cited as integral to efficacy variation (Beebe et al., 2016; Bennett, 2015; Kokocińska‐Kusiak et al., 2021; Lazarowski et al., 2020; Wasser et al., 2004), but the results of how it can alter CDD performance are mixed (Glen & Veltman, 2018). In some cases, detection rates have been seen to have a positive relationship with wind speed (Mutoro et al., 2021). With vegetation density, a weak negative relationship for CDD, but a strong negative relationship for humans (Domínguez del Valle et al., 2020). The effect of vegetation density can also be altered by other elements such as temperature, where Grimm‐Seyfarth (2022) found a negative relationship between detection probability and temperature when searching in short grass, and a positive relationship when searching in tall grass. There are a few proposed explanations for this, such as how vegetation density can alter scent movement (Gutzwiller, 1990), and how higher temperatures can lead to reduced humidity, increased panting rates for the dog (Osterkamp, 2020), increased direct sunlight (Gutzwiller, 1990; MacKay et al., 2008) and higher amounts of flying insects which may deter the CDD (MacKay et al., 2008) or move the scent plume (Osterkamp, 2020), all of which can reduce detection probability. Furthermore, precipitation can be a concern as it can wash away or degrade samples (Reed et al., 2011). In other cases, no effects of temperature, wind speed, humidity, or vegetation were found across studies looking for a range of targets including mammalian carnivore scats, bat and bird carcasses at windfarms, scat from different species of quoll, Hermann tortoises, cheetah scat and bird carcasses infected with avian botulism (Hofmann et al., 2021; Jean‐Marie et al., 2019; Leigh & Dominick, 2015; Long et al., 2007; Mutoro et al., 2021; Paula et al., 2011; Reed et al., 2011; Reynolds et al., 2021; Smith et al., 2005; Thompson et al., 2012). Indeed, it should be noted that not only the environmental conditions themselves, but also how a handler deals with them can have an impact on the search. This includes how the environment affects handler fatigue which in turn impacts the handler's behaviour and body language (Osterkamp, 2020), as well as their ability to keep on‐transect while also focusing on the CDD (MacKay et al., 2008). As such, it is clear that environmental effects can be highly variable, and MacKay et al. (2008) argue that the question on the effect of environmental conditions on CDD searches needs to be given further attention.

Regarding search methods, elements that differ include searching on or off leash, operational time and effective search distance. In terms of how dogs search alongside handlers, it is recommended that CDD perform off‐leash searches to avoid handler bias and allow the dog to move freely and make independent decisions regarding following scent trails (Bennett, 2015; Domínguez del Valle et al., 2020; MacKay et al., 2008). This would mean that those who opt for line search where the dog is leashed may be inadvertently altering efficacy. However, line search must be conducted in some circumstances due to safety concerns for the dog regarding the environment or predators, dense vegetation, or safety for wildlife (DeMatteo et al., 2014; Hansen & Winje, 2021; MacKay et al., 2008). Line searches can also be useful for detailed searches for small odour sources, but not necessary (MacKay et al., 2008; Woollett et al., 2013). Traditionally, operational searches occur in 30‐min intervals (Centre for the Protection of National Infrastructure, 2018), but evidence suggests dogs may be able to work continuously for up to 2 h if so trained (Garner et al., 2001). As such, if the dog has been conditioned poorly for operational searches, they may become demotivated or fatigued too soon into a search which could cause their efficacy to drop. Lastly, CDD have an effective operational search distance from the handler or transect lines. Despite maximum recorded search distances of up to 62.8 m (Cablk et al., 2008), handlers should have continuous visuals of the dog for safety and noticing alerts promptly. In addition, efficacy does appear to be negatively related to search distance (Baker et al., 2021; Reed et al., 2011). Therefore, the recommendation is usually less than 10–15 m for the most efficient and productive search (Glen et al., 2018; Glen & Veltman, 2018; Goodwin et al., 2010), although even this can vary if wind directions and speed are more optimal for the dog which can increase olfaction abilities (Glen et al., 2018).

### 4.4 Future progress

Although there are clearly issues that need to be addressed regarding CDD use, research and efficacy, the benefits CDD can offer to conservation in this time of worldwide ecological crisis demonstrates the necessity to improve their utilisation for the future. Conservation in general requires more funding to achieve its goals and slow down species decline (Malcom et al., 2019). If CDD teams had more financial resources, then the budget constraints which prevent some studies from conducting efficacy assessments or deploying CDD on larger scales would be less of a problem. Furthermore, greater communication between CDD practitioners and researchers across institutions could lead to the development of empirical standards of practice with the subsequent following of standards by authors, researchers and CDD teams globally. To aid in the standardisation of future studies, Table 3 has been produced as a checklist to help guide manuscript authors on what variables to include in their publication.

**TABLE 3:** Checklist of variables that could affect efficacy, and should be included in studies describing training, testing, or operational work of conservation detection dogs (CDD).

Variable	Description and questions to answer in study
Training samples	These are the samples used to train the dog on the target odour. What are they composed of? How many of them were there? How often were they used? How were they stored? How were they handled?
Testing samples	These are the samples used to test the dog's efficacy. Were these different to training samples? If not, why not? What samples were used for discrimination? Were they very similar to the target sample and why were they selected?
Other target odours	Many CDD are used in multiple studies and therefore may have more than one target odour. Have the dogs used been previously trained on other species that could be in that environment?
Odour level	This is a description of the concentration of the odour the dog is trained or tested on i.e. parts per million or size and surface area of sample. Has the dog been trained to find a variety of odour levels, and do those odour levels represent what the dog will be looking for in the field? i.e. dogs that are used to find bats around turbines should be trained to find bat body parts as well as full carcasses as that is what they will be finding in the field.
Indication	The final response trained to show a dog has found something. What indication is the dog using? Is it appropriate for the species being detected? Is the handler able to identify the dog's indication or change of behaviour and what change of behaviour is being used that equates to a positive indication?
Blinding	This describes who is present during training and testing and who knows where the target is during training and testing. Some form of blinding should be used throughout training and testing. During training single blinding can be performed for certain stages, but should be changed to double blinding for the last stages of training, and for testing. If this is not the case, why not?
Dog selection	This describes the dogs used for the study and why they were chosen. Why were the dogs used for this study selected? What are the breeds, personalities, diets, ages and overall health of the dogs selected?
Operational experience of dog and handler team and trainers of the dog team have	This describes the experience of the trainer of the dog, and the operational experience of the dog and handler team. What experience does the trainers of the dog and the dog team have? Have they worked in conservation before? How long has this dog and team been operational? Has the dog had previous finds on other species? Has this handler worked with this dog previously? Is the handler and dog used to working in this environment and have they been trained to do the length of searches asked of them?
Environment	These are environmental variables that should recorded before and during a search. Record the temperature, humidity, wind speed, vegetation density, precipitation before and during the search.
Search	This describes how the search was conducted. How was the search conducted? Was it on or off lead? What was the operational search distance and why was this selected?
Efficacy	This describes if the dog is demonstrating the desired effect of their training, that is, to locate specific species or field signs. Has some measure of sensitivity or precision been measured? If not, why not? Has there been some measure to show that the dog is demonstrating the desired effect of their training?

## 5 CONCLUSION

In conclusion, there can be little doubt based on efficacy rates and comparison with other techniques, that using CDD is an effective and beneficial method for conducting a wide range of conservation work. However, the variation in CDD efficacy reported across studies signifies that substantial longstanding issues with standardisation and methodology within the field that are interfering with the understanding of and use of dogs in conservation. CDD are biological systems, meaning their performance is affected by factors including traits of the dog, training methods, experience of both the dog and handler, variables altering olfactory capabilities and the techniques used during a search as well as the search environment itself. This review has critiqued and described ongoing difficulties facing CDD methodology, namely a lack of detail on dogs, handlers, training, experience and study results and contamination of samples during training and searches. The performance of CDD may vary for numerous reasons and as such a cause cannot be determined in any one case without the relevant information. The question is no longer can CDD work in conservation, but rather what can be done to achieve the highest quality performance, while mitigating error and bias. Highlighting these outstanding problems within the literature can enhance future efforts to standardise and improve the CDD research quality, as until then these issues will overshadow the outstanding abilities of CDD.

## AUTHOR CONTRIBUTIONS

**Beth McKeague:** Conceptualization (equal); writing – original draft (lead); writing – review and editing (equal). **Caroline Finlay:** Conceptualization (equal); supervision (equal); writing – review and editing (equal). **Nicola Rooney:** Supervision (equal); writing – review and editing (equal).

## CONFLICT OF INTEREST STATEMENT

There was no conflict of interest for the authors in this study.

## Data Availability

As this was a review no new data was created in its production. All papers used for analysis are described in Table 2. Data sharing not applicable – no new data generated.

## References

[ece310866-bib-0001] Arandjelovic, M. , Bergl, R. A. , Ikfuingei, R. , Jameson, C. , Parker, M. , & Vigilant, L. (2015). Detection dog efficacy for collecting faecal samples from the critically endangered Cross River gorilla (*Gorilla gorilla diehli*) for genetic censusing. Royal Society Open Science, 2(2), 140423. 10.1098/rsos.140423 26064602 PMC4448817

[ece310866-bib-0002] Arnesen, C. H. , Johnsen, C. B. , Costanzi, J. M. , & Rosell, F. (2020). Canines (*Canis lupus familiaris*) as biodetectors for conservation work: Can they discriminate the rock ptarmigan (*Lagopus muta*) from the willow grouse (*L. lagopus*) in a yes/no task? PLoS One, 15(1), e0228143. 31990940 10.1371/journal.pone.0228143 PMC6986717

[ece310866-bib-0003] Arnesen, C. H. , & Rosell, F. (2021). Pest detection dogs for wood boring longhorn beetles. Scientific Reports, 11(1), 16887. 10.1038/s41598-021-96450-0 34413443 PMC8376989

[ece310866-bib-0004] Arnett, E. B. (2006). A preliminary evaluation on the use of dogs to recover bat fatalities at wind energy facilities. Wildlife Society Bulletin (1973–2006), 34(5), 1440–1445.

[ece310866-bib-0005] Arnot, D. E. , Roper, C. , & Bayoumi, R. A. L. (1993). Digital codes from hypervariable tandemly repeated DNA sequences in the *Plasmodium falciparum* circumsporozoite gene can genetically barcode isolates. Molecular and Biochemical Parasitology, 61(1), 15–24. 10.1016/0166-6851(93)90154-P 8259128

[ece310866-bib-0006] Baker, G. B. , Candy, S. , Robinson, S. , Friend, J. A. , Holdsworth, M. , Jensz, K. , Page, M. , & Algar, D. (2021). Effectiveness of dogs for detecting feral cat scats in wheatbelt reserves of Western Australia. Wildlife Research, 48(8), 690–700. 10.1071/WR20118

[ece310866-bib-0007] Bearman‐Brown, L. E. , Wilson, L. E. , Evans, L. C. , & Baker, P. J. (2020). Comparing non‐invasive surveying techniques for elusive, nocturnal mammals: A case study of the west European hedgehog (*Erinaceus europaeus*). Journal of Vertebrate Biology, 69(3), 20071–20075.

[ece310866-bib-0008] Becker, M. S. , Durant, S. M. , Watson, F. G. R. , Parker, M. , Gottelli, D. , M'soka, J. , Droge, E. , Nyirenda, M. , Schuette, P. , Dunkley, S. , & Brummer, R. (2017). Using dogs to find cats: Detection dogs as a survey method for wide‐ranging cheetah. Journal of Zoology, 302(3), 184–192. 10.1111/jzo.12445

[ece310866-bib-0009] Beckmann, J. P. (2006). Carnivore conservation and search dogs: The value of a novel, non‐invasive technique in the greater Yellowstone ecosystem . In Greater Yellowstone Public Lands: A Century of Discovery, Hard Lessons, and Bright Prospects. Proceedings of the 8th Biennial Scientific Conference on the Greater Yellowstone Ecosystem, pp. 20–26.

[ece310866-bib-0010] Beebe, S. C. , Howell, T. J. , & Bennett, P. C. (2016). Using scent detection dogs in conservation settings: A review of scientific literature regarding their selection. Frontiers in Veterinary Science, 3, 96. 10.3389/fvets.2016.00096 27840815 PMC5083854

[ece310866-bib-0011] Bennett, E. M. (2015). Observations from the use of dogs to undertake carcass searches at wind facilities in Australia. In C. Hull , E. Bennett , E. Stark , I. Smales , J. Lau , & M. Venosta (Eds.), Wind and Wildlife (pp. 113–123). Springer. 10.1007/978-94-017-9490-9_7

[ece310866-bib-0012] Bennett, E. M. , Hauser, C. E. , & Moore, J. L. (2020). Evaluating conservation dogs in the search for rare species. Conservation Biology, 34(2), 314–325. 10.1111/cobi.13431 31696558

[ece310866-bib-0013] Bennett, E. M. , Jamieson, L. T. , Florent, S. N. , Gill, N. , Hauser, C. , & Cristescu, R. (2022). Detection dogs provide a powerful method for conservation surveys. Austral Ecology, 47, 894–901. 10.1111/aec.13162

[ece310866-bib-0014] Bergström, A. , Frantz, L. , Schmidt, R. , Ersmark, E. , Lebrasseur, O. , Girdland‐Flink, L. , Lin, A. T. , Storå, J. , Sjögren, K. G. , Anthony, D. , Antipina, E. , Amiri, S. , Bar‐Oz, G. , Bazaliiskii, V. I. , Bulatović, J. , Brown, D. , Carmagnini, A. , Davy, T. , Fedorov, S. , … Skoglund, P. (2020). Origins and genetic legacy of prehistoric dogs. Science, 370(6516), 557–564. 10.1126/science.aba9572 33122379 PMC7116352

[ece310866-bib-0015] Bijland, L. R. , Bomers, M. K. , & Smulders, Y. M. (2013). Smelling the diagnosis a review on the use of scent in diagnosing. The Netherlands Journal of Medicine, 71(2013), 300–307. 23956311

[ece310866-bib-0016] Bolton, M. , Morgan, G. , Bolton, S. , Bolton, J. , Parmor, S. , & Bambini, L. (2021). Teaching old dogs and young dogs new tricks: Canine scent detection for seabird monitoring. Seabird, 33, 35–52.

[ece310866-bib-0017] Boroski, B. B. , & Oliver, L. (2018). Striving for best standards and practices: Recommendations for optimizing assessment and performance of ecological scent detection dogs. In N. L. Richards (Ed.), Using detection dogs to monitor aquatic ecosystem health and protect aquatic resources (pp. 287–302). Springer International Publishing. 10.1007/978-3-319-77356-8_8

[ece310866-bib-0018] Brook, S. M. , van Coeverden de Groot, P. , Scott, C. , Boag, P. , Long, B. , Ley, R. E. , Reischer, G. H. , Williams, A. C. , Mahood, S. P. , Hien, T. M. , Polet, G. , Cox, N. , & Hai, B. T. (2012). Integrated and novel survey methods for rhinoceros populations confirm the extinction of Rhinoceros sondaicus annamiticus from Vietnam. Biological Conservation, 155, 59–67. 10.1016/j.biocon.2012.06.008

[ece310866-bib-0019] Browne, C. M. , Stafford, K. J. , & Fordham, R. A. (2006). The use of scent‐detection dogs. Irish Veterinary Journal, 59(2), 97–104.

[ece310866-bib-0020] Browne, C. M. , Stafford, K. J. , & Fordham, R. A. (2015). The detection and identification of tuatara and gecko scents by dogs. Journal of Veterinary Behavior, 10(6), 496–503. 10.1016/j.jveb.2015.08.002

[ece310866-bib-0021] Byosiere, S.‐E. , Feng, L. C. , & Rutter, N. J. (2019). Factors that may affect the success of scent detection dogs: Exploring non‐conventional models of preparation and deployment. Comparative Cognition & Behavior Reviews, 14, 81–86. 10.3819/CCBR.2019.140009

[ece310866-bib-0022] Cablk, M. E. , & Heaton, J. S. (2006). Accuracy and reliability of dogs in surveying for desert tortoise (*Gopherus agassizii*). Ecological Applications, 16(5), 1926–1935. 17069383 10.1890/1051-0761(2006)016[1926:aarodi]2.0.co;2

[ece310866-bib-0023] Cablk, M. E. , Sagebiel, J. C. , Heaton, J. S. , & Valentin, C. (2008). Olfaction‐based detection distance: A quantitative analysis of how far away dogs recognize tortoise odor and follow it to source. Sensors, 8(4), 2208–2222. 27879818 10.3390/s8042208 PMC3673414

[ece310866-bib-0024] Cambau, E. , & Poljak, M. (2020). Sniffing animals as a diagnostic tool in infectious diseases. Clinical Microbiology and Infection, 26(4), 431–435. 10.1016/j.cmi.2019.10.036 31734357

[ece310866-bib-0025] Ceballos, G. , Ehrlich, P. R. , & Dirzo, R. (2017). Biological annihilation via the ongoing sixth mass extinction signaled by vertebrate population losses and declines. Proceedings of the National Academy of Sciences of the United States of America, 114(30), E6089–E6096. 10.1073/pnas.1704949114 28696295 PMC5544311

[ece310866-bib-0026] Centre for the Protection of National Infrastructure . (2018). Key points to consider when specifying, procuring and implementing detection dog services . National Protective Security Authority, UK. https://www.npsa.gov.uk/system/files/documents/key‐points‐consider‐when‐specifying‐procuring‐and‐implementing‐detection‐dog‐services.pdf

[ece310866-bib-0027] Chambers, C. L. , Vojta, C. D. , Mering, E. D. , & Davenport, B. (2015). Efficacy of scent‐detection dogs for locating bat roosts in trees and snags. Wildlife Society Bulletin, 39(4), 780–787. 10.1002/wsb.598

[ece310866-bib-0028] Clare, J. D. J. , Anderson, E. M. , MACfarland, D. M. , & Sloss, B. L. (2015). Comparing the costs and detectability of bobcat using scat‐detecting dog and remote camera surveys in central Wisconsin. Wildlife Society Bulletin, 39(1), 210–217. 10.1002/wsb.502

[ece310866-bib-0029] Cozzi, G. , Hollerbach, L. , Suter, S. M. , Reiners, T. E. , Kunz, F. , Tettamanti, F. , & Ozgul, A. (2021). Eyes, ears, or nose? Comparison of three non‐invasive methods to survey wolf recolonisation. Mammalian Biology, 101(6), 881–893.

[ece310866-bib-0030] Cristescu, R. H. , Foley, E. , Markula, A. , Jackson, G. , Jones, D. , & Frère, C. (2015). Accuracy and efficiency of detection dogs: A powerful new tool for koala conservation and management. Scientific Reports, 5(1), 8349. 10.1038/srep08349 25666691 PMC4322364

[ece310866-bib-0031] Cristescu, R. H. , Miller, R. L. , & Frère, C. H. (2020). Sniffing out solutions to enhance conservation: How detection dogs can maximise research and management outcomes, through the example of koalas. Australian Zoologist, 40(3), 416–432. 10.7882/AZ.2019.030

[ece310866-bib-0032] Cristescu, R. H. , Miller, R. L. , Schultz, A. J. , Hulse, L. , Jaccoud, D. , Johnston, S. , Hanger, J. , Booth, R. , & Frère, C. H. (2019). Developing noninvasive methodologies to assess koala population health through detecting *Chlamydia* from scats. Molecular Ecology Resources, 19(4), 957–969. 10.1111/1755-0998.12999 30681773

[ece310866-bib-0033] Davidson, G. A. , Clark, D. A. , Johnson, B. K. , Waits, L. P. , & Adams, J. R. (2014). Estimating cougar densities in northeast Oregon using conservation detection dogs. The Journal of Wildlife Management, 78(6), 1104–1114. 10.1002/jwmg.758

[ece310866-bib-0034] de Oliveira, M. L. , Norris, D. , Ramírez, J. F. M. , Peres, P. H. F. , Galetti, M. , & Duarte, J. M. B. (2012). Dogs can detect scat samples more efficiently than humans: An experiment in a continuous Atlantic Forest remnant. Zoologia (Curitiba Impresso), 29(2), 183–186. 10.1590/S1984-46702012000200012

[ece310866-bib-0035] DeMatteo, K. E. , Blake, L. W. , Young, J. K. , & Davenport, B. (2018). How behavior of nontarget species affects perceived accuracy of scat detection dog surveys. Scientific Reports, 8(1), 13830. 10.1038/s41598-018-32244-1 30218000 PMC6138736

[ece310866-bib-0036] DeMatteo, K. E. , Davenport, B. , & Wilson, L. E. (2019). Back to the basics with conservation detection dogs: Fundamentals for success. Wildlife Biology, 2019(1), 1–9. 10.2981/wlb.00584

[ece310866-bib-0037] DeMatteo, K. E. , Rinas, M. A. , Argüelles, C. F. , Holman, B. E. , Di Bitetti, M. S. , Davenport, B. , Parker, P. G. , & Eggert, L. S. (2014). Using detection dogs and genetic analyses of scat to expand knowledge and assist felid conservation in Misiones, Argentina. Integrative Zoology, 9(5), 623–639. 10.1111/1749-4877.12113 25236691

[ece310866-bib-0038] Dematteo, K. E. , Rinas, M. A. , Sede, M. M. , Davenport, B. , Argüelles, C. F. , Lovett, K. , & Parker, P. G. (2009). Detection dogs: An effective technique for bush dog surveys. Journal of Wildlife Management, 73(8), 1436–1440. 10.2193/2008-545

[ece310866-bib-0039] Díaz, S. , Settele, J. , Brondízio, E. S. , Ngo, H. T. , Guèze, M. , Agard, J. , Arneth, A. , Balvanera, P. , Brauman, K. A. , Butchart, S. H. M. , Chan, K. M. A. , Garibaldi, L. A. , Liu, K. I. J. , Subramanian, S. M. , Midgley, G. F. , Miloslavich, P. , Molnár, Z. , Obura, D. , Pfaff, A. , … Zayas, C. N. (2019). Summary for policymakers of the global assessment report of the intergovernmental science‐policy platform on biodiversity and ecosystem services , Bonn, IPBES Secretariat, 39.

[ece310866-bib-0040] Domínguez del Valle, J. , Cervantes Peralta, F. , & Jaquero, A. M. I. (2020). Factors affecting carcass detection at wind farms using dogs and human searchers. Journal of Applied Ecology, 57(10), 1926–1935. Edited by A. McKenzie. 10.1111/1365-2664.13714

[ece310866-bib-0041] Duggan, J. M. , Heske, E. J. , Schooley, R. L. , Hurt, A. , & Whitelaw, A. (2011). Comparing detection dog and livetrapping surveys for a cryptic rodent. The Journal of Wildlife Management, 75(5), 1209–1217. 10.1002/jwmg.150

[ece310866-bib-0042] Elliker, K. R. , Sommerville, B. A. , Broom, D. M. , Neal, D. E. , Armstrong, S. , & Williams, H. C. (2014). Key considerations for the experimental training and evaluation of cancer odour detection dogs: Lessons learnt from a double‐blind, controlled trial of prostate cancer detection. BMC Urology, 14(1), 22. 10.1186/1471-2490-14-22 24575737 PMC3945616

[ece310866-bib-0043] Engeman, R. M. , Vice, D. S. , York, D. , & Gruver, K. S. (2002). Sustained evaluation of the effectiveness of detector dogs for locating brown tree snakes in cargo outbound from Guam. International Biodeterioration & Biodegradation, 49(2–3), 101–106.

[ece310866-bib-0044] Fukuhara, R. , Agarie, J. , Furugen, M. , & Seki, H. (2022). Nesting habitats of free‐ranging Indian peafowl, *Pavo cristatus*, revealed by sniffer dogs in Okinawa, Japan. Applied Animal Behaviour Science, 249, 105605. 10.1016/j.applanim.2022.105605

[ece310866-bib-0045] Fukuhara, R. , Yamaguchi, T. , Ukuta, H. , Roy, S. , Tanaka, J. , & Ogura, G. (2010). Development and introduction of detection dogs in surveying for scats of small Indian mongoose as invasive alien species. Journal of Veterinary Behavior, 5(2), 101–111. 10.1016/j.jveb.2009.08.010

[ece310866-bib-0046] Furtado, M. M. , Carrillo‐Percastegui, S. E. , Jácomo, A. T. , Powell, G. V. , Silveira, L. , Vynne, C. , & Sollmann, R. (2008). Studying jaguars in the wild: Past experiences and future perspectives. Cat News, 4(Special issue), 41–47.

[ece310866-bib-0047] Furton, K. , Greb, J. , & Holness, H. (2010). The scientific working group on dog and orthogonal detector guidelines (SWGDOG) . National Criminal Justive Reference Service, 155.

[ece310866-bib-0048] Gadbois, S. , & Reeve, C. (2016). The semiotic canine: Scent processing dogs as research assistants in biomedical and environmental research. Dog Behavior, 2(3), 26–32.

[ece310866-bib-0049] Garner, K. J. , Busbee, L. , Cornwell, P. , Edmonds, J. , Mullins, K. , Rader, K. , Johnston, J. M. , & Williams, J. (2001). Duty cycle of the detector dog: A baseline study . Institute for Biological Detection Systems*, Auburn University* [Preprint].

[ece310866-bib-0050] Giardina, E. (2013). DNA fingerprinting. In S. Maloy , & K. T. Hughes (Eds.), Brenner's encyclopedia of genetics (pp. 356–359). Elsevier. 10.1016/B978-0-12-374984-0.00419-8

[ece310866-bib-0051] Glen, A. S. , Anderson, D. , Veltman, C. J. , Garvey, P. M. , & Nichols, M. (2016). Wildlife detector dogs and camera traps: A comparison of techniques for detecting feral cats. New Zealand Journal of Zoology, 43(2), 127–137.

[ece310866-bib-0052] Glen, A. S. , Russell, J. C. , Veltman, C. J. , & Fewster, R. M. (2018). I smell a rat! Estimating effective sweep width for searches using wildlife‐detector dogs. Wildlife Research, 45(6), 500. 10.1071/WR18021

[ece310866-bib-0053] Glen, A. S. , & Veltman, C. J. (2018). Search strategies for conservation detection dogs. Wildlife Biology, 2018(1), 1–9. 10.2981/wlb.00393

[ece310866-bib-0054] Goodwin, K. M. , Engel, R. E. , & Weaver, D. K. (2010). Trained dogs outperform human surveyors in the detection of rare spotted knapweed (*Centaurea stoebe*). Invasive Plant Science and Management, 3(2), 113–121. 10.1614/IPSM-D-09-00025.1

[ece310866-bib-0055] Goss, K.‐U. (2019). The physical chemistry of odors — Consequences for the work with detection dogs. Forensic Science International, 296, 110–114. 10.1016/j.forsciint.2019.01.023 30711845

[ece310866-bib-0056] Grimm‐Seyfarth, A. (2022). Environmental and training factors affect canine detection probabilities for terrestrial newt surveys. Journal of Veterinary Behavior, 57, 6–15. 10.1016/j.jveb.2022.07.013

[ece310866-bib-0057] Grimm‐Seyfarth, A. , Harms, W. , & Berger, A. (2021). Detection dogs in nature conservation: A database on their world‐wide deployment with a review on breeds used and their performance compared to other methods. Methods in Ecology and Evolution. Edited by D. Fisher, 12(4), 568–579. 10.1111/2041-210X.13560

[ece310866-bib-0058] Grimm‐Seyfarth, A. , & Klenke, R. (2018). How to detect elusive species? Detection dogs in nature conservation. *Proceedings of the 5th European congress of conservation biology*. Jyväskylä: Jyvaskyla University Open Science Centre 10.17011/conference/eccb2018/108096

[ece310866-bib-0059] Grimm‐Seyfarth, A. , Zarzycka, A. , Nitz, T. , Heynig, L. , Weissheimer, N. , Lampa, S. , & Klenke, R. (2019). Performance of detection dogs and visual searches for scat detection and discrimination amongst related species with identical diets. Nature Conservation, 37, 81–98. 10.3897/natureconservation.37.48208

[ece310866-bib-0060] Guest, C. M. , Harris, R. , Anjum, I. , Concha, A. R. , & Rooney, N. J. (2020). A lesson in standardization – Subtle aspects of the processing of samples can greatly affect dogs' learning. Frontiers in Veterinary Science, 7, 525. 10.3389/fvets.2020.00525 33015138 PMC7461772

[ece310866-bib-0061] Gutzwiller, K. J. (1990). Minimizing dog‐induced biases in game bird research. Wildlife Society Bulletin (1973–2006), 18(3), 351–356.

[ece310866-bib-0062] Hall, N. J. , Glenn, K. , Smith, D. W. , & Wynne, C. D. L. (2015). Performance of pugs, German shepherds, and greyhounds (*Canis lupus familiaris*) on an odor‐discrimination task. Journal of Comparative Psychology, 129(3), 237–246. 26010195 10.1037/a0039271

[ece310866-bib-0063] Hansen, I. , & Winje, E. (2021). Efficiency of livestock carcass detection dogs. Rangelands, 43(5), 194–199. 10.1016/j.rala.2021.03.004

[ece310866-bib-0064] Harrison, R. L. (2006). A comparison of survey methods for detecting bobcats. Wildlife Society Bulletin (1973–2006), 34(2), 548–552.

[ece310866-bib-0065] Hatlauf, J. , Böcker, F. , Wirk, L. , Collet, S. , Schley, L. , Szabó, L. , Hackländer, K. , & Heltai, M. (2021). Jackal in hide: Detection dogs show first success in the quest for golden jackal (*Canis aureus*) scats. Mammal Research, 66(1), 227–236. 10.1007/s13364-020-00537-4

[ece310866-bib-0066] Hayes, J. E. , McGreevy, P. D. , Forbes, S. L. , Laing, G. , & Stuetz, R. M. (2018). Critical review of dog detection and the influences of physiology, training, and analytical methodologies. Talanta, 185, 499–512. 10.1016/j.talanta.2018.04.010 29759233

[ece310866-bib-0067] Heaton, J. S. , Cablk, M. E. , Nussear, K. E. , Esque, T. C. , Medica, P. A. , Sagebiel, J. C. , & Francis, S. S. (2008). Comparison of effects of humans versus wildlife‐detector dogs. The Southwestern Naturalist, 53(4), 472–479.

[ece310866-bib-0068] Helton, W. S. (2009). Canine ergonomics: The science of working dogs. CRC Press.

[ece310866-bib-0069] Hill, S. , & Hill, J. (1987). Richard Henry of Resolution Island. Cadsonbury Publications. John McIndoe.

[ece310866-bib-0070] Hofmann, T. , Marker, L. , & Hondong, H. (2021). Detection success of cheetah (*Acinonyx jubatus*) scat by dog‐human and human‐only teams in a semi‐arid savanna. Namibian Journal of Environment, 5, p. A‐, 1–11.

[ece310866-bib-0071] Hollerbach, L. , Heurich, M. , Reiners, T. E. , & Nowak, C. (2018). Detection dogs allow for systematic non‐invasive collection of DNA samples from Eurasian lynx. Mammalian Biology, 90, 42–46. 10.1016/j.mambio.2018.02.003

[ece310866-bib-0072] Hoyer‐Tomiczek, U. , Sauseng, G. , & Hoch, G. (2016). Scent detection dogs for the Asian longhorn beetle, *Anoplophora glabripennis* . EPPO Bulletin, 46(1), 148–155.

[ece310866-bib-0073] Jamieson, L. T. J. , Baxter, G. S. , & Murray, P. J. (2017). Identifying suitable detection dogs. Applied Animal Behaviour Science, 195, 1–7. 10.1016/j.applanim.2017.06.010

[ece310866-bib-0074] Jamieson, L. T. J. , Baxter, G. S. , & Murray, P. J. (2018a). Who's a good handler? Important skills and personality profiles of wildlife detection dog handlers. Animals, 8(12), 222. 10.3390/ani8120222 30486469 PMC6316394

[ece310866-bib-0075] Jamieson, L. T. J. , Baxter, G. S. , & Murray, P. J. (2018b). You are not my handler! Impact of changing handlers on dogs' Behaviours and detection performance. Animals, 8(10), 176. 10.3390/ani8100176 30304841 PMC6211013

[ece310866-bib-0076] Jean‐Marie, B. , Raphael, G. , Fabien, R. , Aurélien, B. , Sébastien, C. , Nicolas, B. , & Xavier, B. (2019). Excellent performances of dogs to detect cryptic tortoises in Mediterranean scrublands. Biodiversity and Conservation, 28(14), 4027–4045. 10.1007/s10531-019-01863-z

[ece310866-bib-0077] Jenkins, E. K. , DeChant, M. T. , & Perry, E. B. (2018). When the nose Doesn't know: Canine olfactory function associated with health, management, and potential links to microbiota. Frontiers in Veterinary Science, 5, 56. 10.3389/fvets.2018.00056 29651421 PMC5884888

[ece310866-bib-0078] Jezierski, T. , Ensminger, J. , & Papet, L. E. (2016). Canine olfaction science and law: Advances in forensic science, medicine, conservation, and environmental remediation. CRC Press.

[ece310866-bib-0079] Johnen, D. , Heuwieser, W. , & Fischer‐Tenhagen, C. (2017). An approach to identify bias in scent detection dog testing. Applied Animal Behaviour Science, 189, 1–12.

[ece310866-bib-0080] Kapfer, J. M. , Munoz, D. J. , & Tomasek, T. (2012). Use of wildlife detector dogs to study eastern box turtle (*Terrapene carolina carolina*) populations. Herpetological Conservation and Biology, 7(2), 169–175.

[ece310866-bib-0081] Kardish, M. R. , Mueller, U. G. , Amador‐Vargas, S. , Dietrich, E. I. , Ma, R. , Barrett, B. , & Fang, C. C. (2015). Blind trust in unblinded observation in ecology, evolution, and behavior. Frontiers in Ecology and Evolution, 3, 51. 10.3389/fevo.2015.00051

[ece310866-bib-0082] Kerley, L. L. (2010). Using dogs for tiger conservation and research. Integrative Zoology, 5(4), 390–396. 10.1111/j.1749-4877.2010.00217.x 21392356

[ece310866-bib-0083] Kokocińska‐Kusiak, A. , Woszczyło, M. , Zybala, M. , Maciocha, J. , Barłowska, K. , & Dzięcioł, M. (2021). Canine olfaction: Physiology, behavior, and possibilities for practical applications. Animals, 11(8), 2463. 10.3390/ani11082463 34438920 PMC8388720

[ece310866-bib-0084] Kretser, H. , Glennon, M. , Whitelaw, A. , Hurt, A. , Pilgrim, K. , & Schwartz, M. (2016). Scat‐detection dogs survey low density moose in New York. Alces, 52, 55–66.

[ece310866-bib-0085] Lazarowski, L. , Krichbaum, S. , DeGreeff, L. , Simon, A. , Singletary, M. , Angle, C. , & Waggoner, L. P. (2020). Methodological considerations in canine olfactory detection research. Frontiers in Veterinary Science, 7, 408. 10.3389/fvets.2020.00408 32766296 PMC7379233

[ece310866-bib-0086] Lazarowski, L. , Rogers, B. , Waggoner, L. P. , & Katz, J. S. (2019). When the nose knows: Ontogenetic changes in detection dogs' (*Canis familiaris*) responsiveness to social and olfactory cues. Animal Behaviour, 153, 61–68.

[ece310866-bib-0087] Leigh, K. A. , & Dominick, M. (2015). An assessment of the effects of habitat structure on the scat finding performance of a wildlife detection dog. Methods in Ecology and Evolution. Edited by J. McPherson, 6(7), 745–752. htt10.1111/2041‐210X.12374

[ece310866-bib-0088] Liczner, A. R. , MacPhail, V. , Woollett, D. A. , Richards, N. L. , & Colla, S. R. (2021). Training and usage of detection dogs to better understand bumble bee nesting habitat: Challenges and opportunities. PLoS One. Edited by G. Smagghe, 16(5), e0249248. 10.1371/journal.pone.0249248 33979352 PMC8115777

[ece310866-bib-0089] Lit, L. , Schweitzer, J. B. , & Oberbauer, A. M. (2011). Handler beliefs affect scent detection dog outcomes. Animal Cognition, 14(3), 387–394. 10.1007/s10071-010-0373-2 21225441 PMC3078300

[ece310866-bib-0090] Long, R. A. , Donovan, T. M. , Mackay, P. , Zielinski, W. J. , & Buzas, J. S. (2007). Comparing scat detection dogs, cameras, and hair snares for surveying carnivores. Journal of Wildlife Management, 71(6), 2018–2025. 10.2193/2006-292

[ece310866-bib-0091] MacKay, P. , Zielinski, W. J. , Long, R. A. , & Ray, J. C. (2008). Scat detection dogs. In R. A. Long , P. MacKay , J. C. Ray , & W. J. Zielinski (Eds.), Noninvasive survey methods for carnivores (pp. 183–222). Island Press.

[ece310866-bib-0092] Malcom, J. , Schwartz, M. W. , Evansen, M. , Ripple, W. J. , Polasky, S. , Gerber, L. R. , Lovejoy, T. E. , Talbot, L. M. , Miller, J. R. B. , & 1648 signatories . (2019). Solve the biodiversity crisis with funding. Science. Edited by J. Sills, 365(6459), 1256. 10.1126/science.aay9839 31604231

[ece310866-bib-0093] Mathews, F. , Swindells, M. , Goodhead, R. , August, T. A. , Hardman, P. , Linton, D. M. , & Hosken, D. J. (2013). Effectiveness of search dogs compared with human observers in locating bat carcasses at wind‐turbine sites: A blinded randomized trial. Wildlife Society Bulletin, 37(1), 34–40. 10.1002/wsb.256

[ece310866-bib-0094] Matthew, E. E. , Verster, R. , & Weldon, C. (2021). A case study in canine detection of giant bullfrog scent. Journal of Vertebrate Biology, 69(3), 20041–20043.

[ece310866-bib-0095] McGregor, H. W. , Hampton, J. O. , Lisle, D. , & Legge, S. (2016). Live‐capture of feral cats using tracking dogs and darting, with comparisons to leg‐hold trapping. Wildlife Research, 43(4), 313. 10.1071/WR15134

[ece310866-bib-0096] Merriam‐Webster.com Dictionary . (2023). *Efficacy*, *Merriam‐Webster* . https://www.merriam‐webster.com/dictionary/efficacy

[ece310866-bib-0097] Mosconi, F. , Campanaro, A. , Carpaneto, G. M. , Chiari, S. , Hardersen, S. , Mancini, E. , Maurizi, E. , Sabatelli, S. , Zauli, A. , Mason, F. , & Audisio, P. (2017). Training of a dog for the monitoring of *Osmoderma eremita* . Nature Conservation, 20, 237–264. 10.3897/natureconservation.20.12688

[ece310866-bib-0098] Mutoro, N. M. , Eberle, J. , Petermann, J. S. , Schaab, G. , Wykstra, M. , & Habel, J. C. (2021). Impact of weather conditions on cheetah monitoring with scat detection dogs. Journal of Tropical Ecology, 37(5), 222–227. 10.1017/S0266467421000316

[ece310866-bib-0099] Needs, S. , Bennett, E. , Mao, B. , & Hauser, C. E. (2021). Do detection dogs respond differently to dried, frozen and live plant targets? Applied Animal Behaviour Science, 236, 105276. 10.1016/j.applanim.2021.105276

[ece310866-bib-0100] O'Connor, S. , Park, K. J. , & Goulson, D. (2012). Humans versus dogs; a comparison of methods for the detection of bumble bee nests. Journal of Apicultural Research, 51(2), 204–211. 10.3896/IBRA.1.51.2.09

[ece310866-bib-0101] Oh, Y. , Lee, Y. , Heath, J. , & Kim, M. (2015). Applications of animal biosensors: A review. IEEE Sensors Journal, 15(2), 637–645. 10.1109/JSEN.2014.2358261

[ece310866-bib-0102] Oldenburg, C. , Schoon, A. , & Heitkönig, I. M. A. (2016). Wildlife detection dog training: A case study on achieving generalization between target odor variations while retaining specificity. Journal of Veterinary Behavior, 13, 34–38. 10.1016/j.jveb.2016.03.008

[ece310866-bib-0103] Orkin, J. D. , Yang, Y. , Yang, C. , Yu, D. W. , & Jiang, X. (2016). Cost‐effective scat‐detection dogs: Unleashing a powerful new tool for international mammalian conservation biology. Scientific Reports, 6(1), 34758. 10.1038/srep34758 27721442 PMC5056371

[ece310866-bib-0104] Osterkamp, T. (2020). Detector dogs and scent movement. CRC Press. 10.4324/9780429020704

[ece310866-bib-0105] Otto, C. M. , Cobb, M. L. , & Wilsson, E. (2019). Editorial: Working dogs: Form and function. Frontiers in Veterinary Science, 6, 351. 10.3389/fvets.2019.00351 31681808 PMC6813466

[ece310866-bib-0106] Paula, J. , Leal, M. C. , Silva, M. J. , Mascarenhas, R. , Costa, H. , & Mascarenhas, M. (2011). Dogs as a tool to improve bird‐strike mortality estimates at wind farms. Journal for Nature Conservation, 19(4), 202–208. 10.1016/j.jnc.2011.01.002

[ece310866-bib-0107] Petroelje, T. R. , Fowler, N. L. , Kautz, T. M. , Lutto, A. L. , Davidson, G. A. , Beyer, D. E., Jr. , & Belant, J. L. (2021). Conservation detection dogs increase efficacy for prey detection at carnivore GPS cluster sites during summer. Wildlife Society Bulletin, 45(3), 402–409. 10.1002/wsb.1203

[ece310866-bib-0108] Reed, S. E. , Bidlack, A. L. , Hurt, A. , & Getz, W. M. (2011). Detection distance and environmental factors in conservation detection dog surveys. The Journal of Wildlife Management, 75(1), 243–251. 10.1002/jwmg.8

[ece310866-bib-0109] Reindl‐Thompson, S. A. , Shivik, J. A. , Whitelaw, A. , Hurt, A. , & Higgins, K. F. (2006). Efficacy of scent dogs in detecting black‐footed ferrets at a reintroduction site in South Dakota. Wildlife Society Bulletin, 34(5), 1435–1439.

[ece310866-bib-0110] Reynolds, M. H. , Johnson, K. N. , Schvaneveldt, E. R. , Dewey, D. L. , Uyehara, K. J. , & Hess, S. C. (2021). Efficacy of detection canines for avian botulism surveillance and mitigation. Conservation Science and Practice, 3(6), e397.

[ece310866-bib-0111] Richards, N. L. (Ed.). (2018). Looking ahead: Future directions and considerations for using detection dogs in aquatic environments and ecosystems. Using detection dogs to monitor aquatic ecosystem health and protect aquatic resources (pp. 303–317). Springer International Publishing. 10.1007/978-3-319-77356-8_9

[ece310866-bib-0112] Rolland, R. M. , Hamilton, P. K. , Kraus, S. D. , Davenport, B. , Gillett, R. M. , Rolland, R. M. , Wasser, S. K. , & Kraus, S. D. (2007). Faecal sampling using detection dogs to study reproduction and health in North Atlantic right whales (*Euhalaena glacialis*). Journal of Cetacean Research and Management, 8(2), 121.

[ece310866-bib-0113] Rooney, N. J. , Guest, C. M. , Swanson, L. C. M. , & Morant, S. V. (2019). How effective are trained dogs at alerting their owners to changes in blood glycaemic levels? Variations in performance of glycaemia alert dogs. PLoS One, 14(1), e0210092. 10.1371/journal.pone.0210092 30645613 PMC6333402

[ece310866-bib-0114] Rutter, N. J. , Howell, T. J. , Stukas, A. A. , Pascoe, J. H. , & Bennett, P. C. (2021a). Can volunteers train their pet dogs to detect a novel odor in a controlled environment in under 12 weeks? Journal of Veterinary Behavior, 43, 54–65. 10.1016/j.jveb.2020.09.004

[ece310866-bib-0115] Rutter, N. J. , Howell, T. J. , Stukas, A. A. , Pascoe, J. H. , & Bennett, P. C. (2021b). Diving in nose first: The influence of unfamiliar search scale and environmental context on the search performance of volunteer conservation detection dog–Handler Teams. Animals, 11(4), 1177. 10.3390/ani11041177 33924219 PMC8074607

[ece310866-bib-0116] Rutter, N. J. , Mynott, J. H. , Howell, T. J. , Stukas, A. A. , Pascoe, J. H. , Bennett, P. C. , & Murphy, N. P. (2021). Buzzing with possibilities: Training and olfactory generalization in conservation detection dogs for an endangered stonefly species. Aquatic Conservation: Marine and Freshwater Ecosystems, 31(4), 984–989. 10.1002/aqc.3531

[ece310866-bib-0117] Sebeok, T. A. , & Rosenthal, R. E. (1981). The clever Hans phenomenon: Communication with horses, whales, apes, and people. Annals of the New York Academy of Sciences, 364, 309. 6942738

[ece310866-bib-0118] Sentilles, J. , Vanpé, C. , & Quenette, P.‐Y. (2021). Benefits of incorporating a scat‐detection dog into wildlife monitoring: A case study of Pyrenean brown bear. Journal of Vertebrate Biology, 69(3), 20091–20096.

[ece310866-bib-0119] Shields, J. , & Austin, L. M. (2018). Monitoring invasive and threatened aquatic amphibians, mammals, and birds. In N. Richards (Ed.), Using detection dogs to monitor aquatic ecosystem health and protect aquatic resources (pp. 71–117). Springer International Publishing. 10.1007/978-3-319-77356-8_3

[ece310866-bib-0120] Smallwood, K. S. , Bell, D. A. , & Standish, S. (2020). Dogs detect larger wind energy effects on bats and birds. The Journal of Wildlife Management, 84(5), 852–864.

[ece310866-bib-0121] Smith, D. A. , Ralls, K. , Cypher, B. L. , & Maldonado, J. E. (2005). Assessment of scat‐detection dog surveys to determine kit fox distribution. Wildlife Society Bulletin (1973–2006), 33(3), 897–904.

[ece310866-bib-0122] Smith, D. A. , Ralls, K. , Hurt, A. , Adams, B. , Parker, M. , Davenport, B. , Smith, M. C. , & Maldonado, J. E. (2003). Detection and accuracy rates of dogs trained to find scats of San Joaquin kit foxes (*Vulpes macrotis mutica*). Animal Conservation, 6(4), 339–346. 10.1017/S136794300300341X

[ece310866-bib-0123] Springer, K. (2011). Planning processes for eradication of multiple pest species on Macquarie Island–an Australian case study . Island invasives: Eradication and management. IUCN, Gland, Switzerland, pp. 228–232.

[ece310866-bib-0124] Stanhope, K. , & Sloan, V. (2019). Proposed method for testing and accreditation of great crested newt detection dogs. CIEEM in Practice, 105, 36–40.

[ece310866-bib-0125] Statham, M. J. , (Smith) Woollett, D. A. , Fresquez, S. , Pfeiffer, J. , Richmond, J. , Whitelaw, A. , Richards, N. L. , Westphal, M. F. , & Sacks, B. N. (2020). Noninvasive identification of Herpetofauna: Pairing conservation dogs and genetic analysis. The Journal of Wildlife Management, 84(1), 66–74. 10.1002/jwmg.21772

[ece310866-bib-0126] Stevenson, D. J. , Ravenscroft, K. R. , Zappalorti, R. T. , Ravenscroft, M. D. , Weigley, S. W. , & Jenkins, C. L. (2010). Using a wildlife detector dog for locating eastern indigo snakes (*Drymarchon couperi*). Herpetological Review, 41(4), 437–442.

[ece310866-bib-0127] Sutherland, W. J. , Dicks, L. V. , Petrovan, S. O. , & Smith, R. K. (2021). What works in conservation: 2021. Open Book Publishers.

[ece310866-bib-0128] Teodoro‐Morrison, T. , Diamandis, E. P. , Rifai, N. , Weetjens, B. J. C. , Pennazza, G. , de Boer, N. K. , & Bomers, M. K. (2014). Animal olfactory detection of disease: Promises and pitfalls. Clinical Chemistry, 60(12), 1473–1479. 10.1373/clinchem.2014.231282 25274554

[ece310866-bib-0129] Thomas, M. L. , Baker, L. , Beattie, J. R. , & Baker, A. M. (2020). Determining the efficacy of camera traps, live capture traps, and detection dogs for locating cryptic small mammal species. Ecology and Evolution, 10(2), 1054–1068. 10.1002/ece3.5972 32015864 PMC6988557

[ece310866-bib-0130] Thompson, C. M. , Royle, J. A. , & Garner, J. D. (2012). A framework for inference about carnivore density from unstructured spatial sampling of scat using detector dogs. The Journal of Wildlife Management, 76(4), 863–871. 10.1002/jwmg.317

[ece310866-bib-0131] Thompson, S. A. , Thompson, G. G. , Withers, P. C. , & Bennett, E. M. (2020). Conservation detection dog is better than human searcher in finding bilby (*Macrotis lagotis*) scats. Australian Zoologist, 41(1), 86–93. 10.7882/AZ.2020.012

[ece310866-bib-0132] Vesely, D. (2008). Training of conservation detection dogs to locate Kincaid's lupine (Lupinus sulphureus ssp. kincaidii) . USFWS Report. U.S. Fish & Wildlife Service, Oregon State Office. Oregon Wildlife Institute, Corvallis, Oregon [Preprint].

[ece310866-bib-0133] Vynne, C. , Skalski, J. R. , Machado, R. B. , Groom, M. J. , Jácomo, A. T. , Marinho‐Filho, J. , Ramos Neto, M. B. , Pomilla, C. , Silveira, L. , Smith, H. , & Wasser, S. K. (2011). Effectiveness of scat‐detection dogs in determining species presence in a tropical savanna landscape. Conservation Biology, 25(1), 154–162. 21029162 10.1111/j.1523-1739.2010.01581.x

[ece310866-bib-0134] Wasser, S. K. , Davenport, B. , Ramage, E. R. , Hunt, K. E. , Parker, M. , Clarke, C. , & Stenhouse, G. (2004). Scat detection dogs in wildlife research and management: Application to grizzly and black bears in the Yellowhead ecosystem, Alberta, Canada. Canadian Journal of Zoology, 82(3), 475–492. 10.1139/z04-020

[ece310866-bib-0135] Wasser, S. K. , Hayward, L. S. , Hartman, J. , Booth, R. K. , Broms, K. , Berg, J. , Seely, E. , Lewis, L. , & Smith, H. (2012). Using detection dogs to conduct simultaneous surveys of northern spotted (*Strix occidentalis caurina*) and barred owls (*Strix varia*). PLoS One. Edited by S.A. Rands, 7(8), e42892. 10.1371/journal.pone.0042892 22916175 PMC3419739

[ece310866-bib-0136] Waters, J. , O'Connor, S. , Park, K. J. , & Goulson, D. (2011). Testing a detection dog to locate bumblebee colonies and estimate nest density. Apidologie, 42(2), 200–205. 10.1051/apido/2010056

[ece310866-bib-0137] Whitehouse‐Tedd, K. , Richards, N. , & Parker, M. (2021). Dogs and conservation: Emerging themes and considerations. Journal of Vertebrate Biology, 69(3), 1–4. 10.25225/jvb.E2004

[ece310866-bib-0138] Willcox, D. , Nash, H. C. , Trageser, S. , Kim, H. J. , Hywood, L. , Connelly, E. , Ichu Ichu, G. , Kambale Nyumu, J. , Mousset Moumbolou, C. L. , Ingram, D. J. , & Challender, D. W. S. (2019). Evaluating methods for detecting and monitoring pangolin (Pholidata: Manidae) populations. Global Ecology and Conservation, 17, e00539. 10.1016/j.gecco.2019.e00539

[ece310866-bib-0139] Witherington, B. , Peruyero, P. , Smith, J. R. , MacPhee, M. , Lindborg, R. , Neidhardt, E. , & Savage, A. (2017). Detection dogs for sea turtle Nesting Beach monitoring, management, and conservation outreach. Marine Turtle Newsletter, 152, 1–4.

[ece310866-bib-0140] Woollett, D. A. , Hurt, A. , & Richards, N. L. (2013). The current and future roles of free‐ranging detection dogs in conservation efforts. In M. E. Gompper (Ed.), Free‐ranging dogs and wildlife conservation (pp. 239–264). Oxford.

